# Synthesis of Tetrasubstituted Alkenes via Metathesis

**DOI:** 10.3390/molecules17033348

**Published:** 2012-03-15

**Authors:** Seung-Mann Paek

**Affiliations:** College of Pharmacy, Research Institute of Pharmaceutical Sciences, Gyeongsang National University, Jinju daero, Jinju, Gyeongnam 660-751, Korea; Email: million@gnu.ac.kr; Tel.: +82-55-772-8284; Fax: +82-55-772-8269

**Keywords:** tetrasubstituted alkene, metathesis, catalyst

## Abstract

Fully substituted olefin generation via metathesis is presented. Catalyst development, optimization of reaction conditions and substrate screening are included. In addition, asymmetric alkene metathesis, the cross metathesis reaction for this transformation and its application in natural products will be discussed.

## 1. Introduction

**Scheme 1:**
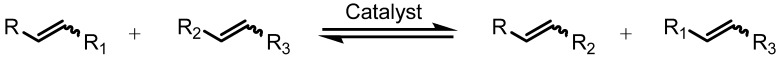
Metathesis reaction and its basic transformation.

**Figure 1:**
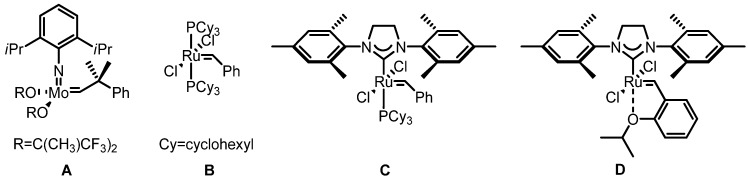
Representative structures of metathesis catalysts.

As an alternative technology for olefin generation, the metathesis reaction has been focused on since its recent and rapid development [1,2]. This transformation allows the efficient production of otherwise readily unavailable olefins including medium-sized heterocycles, carbocycles, trisubstituted olefins and heteroatom-substituted olefins (Scheme 1) using structurally diverse catalysts (Figure 1) [3,4]. Nowadays, the protocol is widely utilized to synthesize valuable polymers, natural products and medicinal compounds for humans [5,6,7].

**Figure 2:**
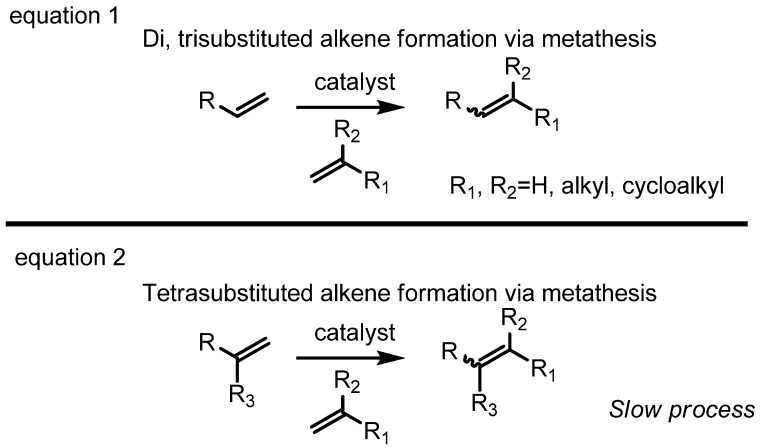
Difficulty in the generation of tetrasubstituted olefins via metathesis.

Although metathesis is a fascinating technology for alkene synthesis, this reaction still needs improvement in terms of reactivity and selectivity [1]. The general pattern that the more substituted a substrate is, the less reactive it is, shows that steric bulkiness hampers the incorporation of metal carbenoids in the substrate. For the selectivity issue of alkene geometry, various methodologies have been developed to control the *E* or *Z* geometry of the resulting olefins [8,9,10,11,12]. However, the general problems of *E-* or *Z*-selective alkene metathesis haven’t yet been solved when it comes to the case of tetrasubstituted olefins (Figure 2).

For the reasons mentioned above, a great effort has already been devoted to the efficient generation of tetrasubsituted alkenes via metathesis. A variety of catalysts, reaction conditions including solvents, substrate screening and applications have been tried. This article presents an overview of these endeavors.

## 2. Results and Discussion

### 2.1. Early Studies with a Molybdenum Catalyst

**Scheme 2:**
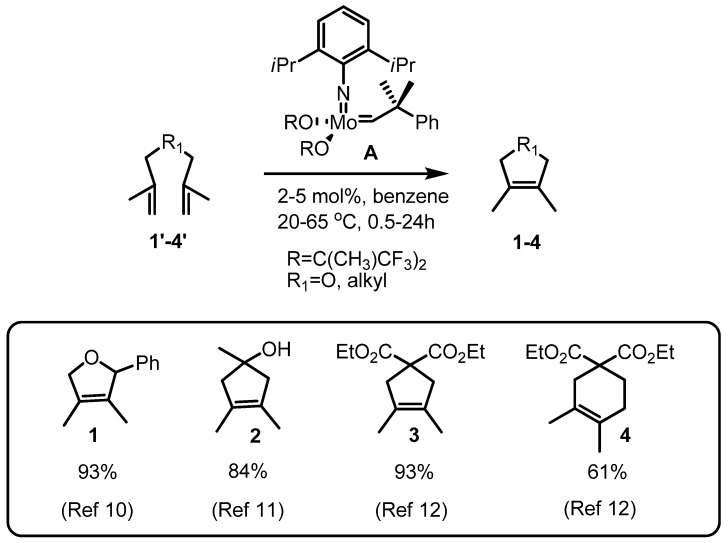
Early utilization of a Mo catalyst **A** in tetrasubstituted olefins.

Actually, tetrasubstituted alkene generation via metathesis was first reported two decades ago, when the rapid ring closing metathesis (RCM) of the substrate to form the sterically encumbered medium-sized ring skeletons **1**–**4** using a highly reactive molybdenum catalyst (**A**) was described (Scheme 2) [13,14,15]. These results indicate that the molybdenum carbenoid can be incorporated into the sterically hindered substrates easily. However, this molybdenum metal species was soon replaced by the ruthenium catalysts **B**–**D**, because of their air and moisture stability and wide utility. Consequently, tetrasubstituted alkene generation via ruthenium catalysts was also studied.

### 2.2. Early Studies with a Ruthenium Catalyst

**Scheme 3:**
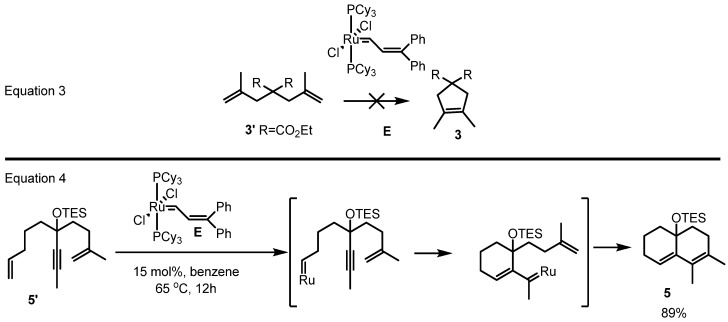
Unsuccessful RCM and successful relay RCM.

The most challenging obstacle to the utilization of ruthenium carbenoids to produce tetrasubstituted alkenes was the reactivity of the ruthenium species. Unlike the molybdenum species **A** which catalyzed the transformation of the diene **3'** into the corresponding product **3** in high yield, the ruthenium catalyst **E** couldn’t perform the same role (Scheme 2 and Equation 3 in Scheme 3) [16].

Considering that the relay-RCM of dienyne **5'** to the corresponding diene product **5** was reported using the same catalyst **E**, the incorporation of the ruthenium carbenoid into the substrate was regarded as a rate determining step in this process (Scheme 3). Definitely, more reactive ruthenium catalysts were necessary. 

### 2.3. New Catalyst Design and Its Application

**Scheme 4:**
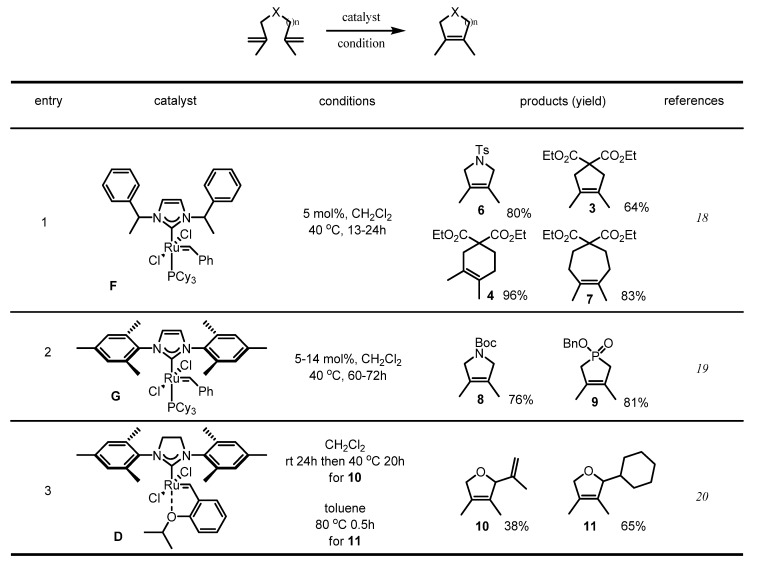
Development of new catalysts and their application.

As is well known, dramatic improvement of ruthenium catalyst reactivity was achieved by replacing the tricyclohexylphosphine (PCy_3_) ligand with an N-heterocyclic carbine (NHC) ligand [17]. Actually, it was reported that a similar ruthenium catalyst **F** with an imidazolylidene ligand could produce tetrasubstituted cycloalkenes or pyrroline products (Entry 1, Scheme 4) [18]. Furthermore, the same imidazolylidene with a more sterically demanding dimesityl group was introduced to catalyze the medium-sized RCM of tetrasubstituted alkenes (Entry 2, Scheme 4) [19]. The Grubbs-Hoveyda catalyst **D** also showed a high performance in producing furans **10** and **11** (Entry 3, Scheme 4) [20].

### 2.4. Optimization of Reaction Conditions

**Scheme 5:**
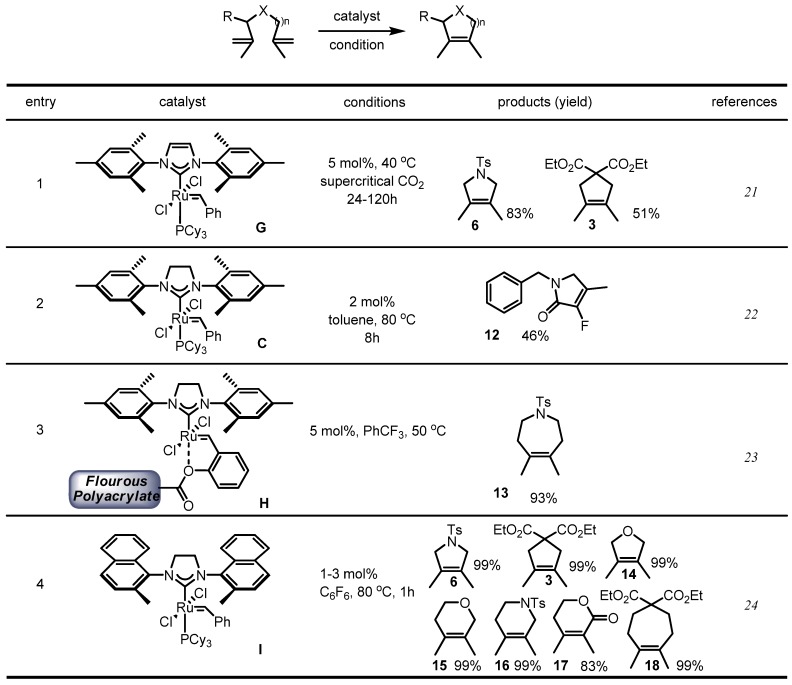
Optimization of the metathesis reaction in tetrasubstituted alkenes.

In addition to catalyst development, other reaction conditions such as solvents and substrate were optimized (Scheme 5). In 2001, Furstner *et al.* utilized supercritical CO_2_ to improve the RCM procedure. This unique condition showed improved results for the fully substituted alkenes **6** and **3** [21]. Another research carried out studied the substrate. Using the established Grubbs 2nd generation catalyst, Haufe *et al.* achieved efficient RCM of a fluoride-substituted alkene **12**. Due to the biological importance of fluoride-containing molecules, this paper was highlighted and later applied to the synthesis of other natural products (Scheme 10, *vide infra*) [22]. A polymer-supported metathesis catalyst **H** was also utilized for this type of conversion. An azepine structure **13** could be constructed using the polymer-supported catalyst **H**, which could be reused [23]. More dramatic advances were achieved when Blecher *et al.* changed solvents from normal benzene to hexafluorobenzene. This solvent change allowed the highly efficient RCM of medium-sized heterocycles in high yield [24].

After optimizing reaction conditions, a large improvement in the tetrasubstituted olefin metathesis reaction was achieved. However, this type of RCM still needed generality for various substrates.

### 2.5. Further Catalyst Development

**Scheme 6:**
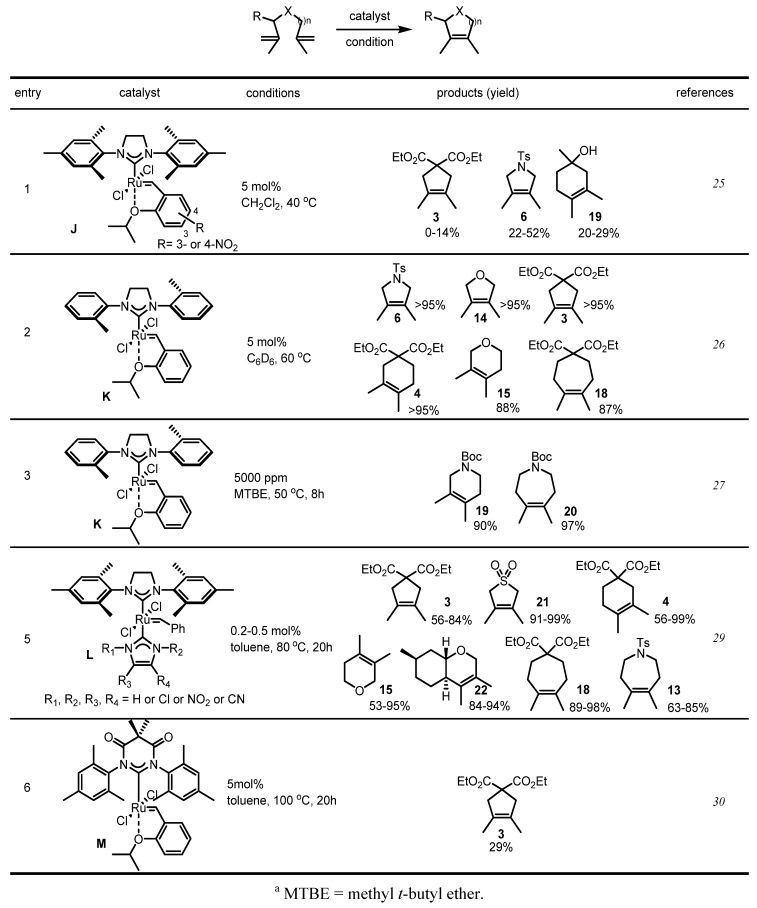
Development of more advanced catalysts **J**–**M**.

More advanced catalyst development research began with the Grubbs-Hoveyda catalyst **D**. Grela *et al.* reported that the NO_2_-substituted aromatic ligand activates the catalyst **J** in the tetrasubstituted olefin metathesis reaction (Entry 1, Scheme 6) [25]. It seems that the electronic effect facilitates the insertion of olefin in the ruthenium carbenoid. However, a more outstanding advance was achieved with the sterically less-demanding liganded catalyst **K**.

Replacement of the mesityl substituent of the toluene moiety made the catalyst **K** much more reactive than the original catalyst **D** [26]. Actually, the catalyst **K** was so reactive that it was sufficient to produce azacycles **19** and **20** at low catalyst loading (Entry 3) [27]. Due to this high performance, the catalyst **K** was used by Stoltz *et al.* to synthesize elatol, a bioactive natural product [28].

Research on catalyst optimization is still ongoing. In 2010, Plenio *et al.* reported the bis-NHC liganded catalyst **L**. In particular, this electron-deficient ligand helped the catalyst to produce various tetrasubstituted olefins by RCM (Entry 5, Scheme 6) [29]. A 6-membered ring ligand was also reported recently. Substitution of imidazoline on the pymidine ligand also showed potential RCM in fully substituted olefins **3** (Entry 6) [30]. Although its chemical yield is relatively low, it is an impressive result as it provides possibilities for ligand modification.

### 2.6. Asymmetric RCM in Tetrasubstituted Alkenes

**Scheme 7:**
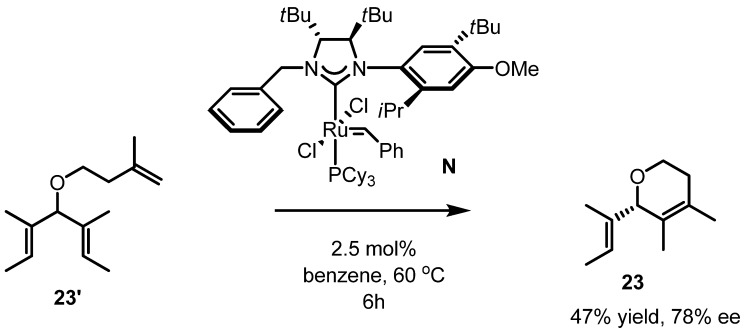
Asymmetric RCM of **23'** to **23**.

As seen in Scheme 7, an experiment on asymmetric RCM was also investigated. Collins *et al.* tried to utilize the metathesis reaction for the introduction of tetrasubstituted asymmetric alkenes. After a set of ligand screens, desymmetrization of the triene **23'** to the optically active dihydropyran **23** could be carried out successfully. It was also reported that the optically active dihydrofuran skeleton could be produced through the same desymmetrization reaction [31]. 

### 2.7. Cross Metathesis in Tetrasubstituted Alkenes

**Scheme 8:**
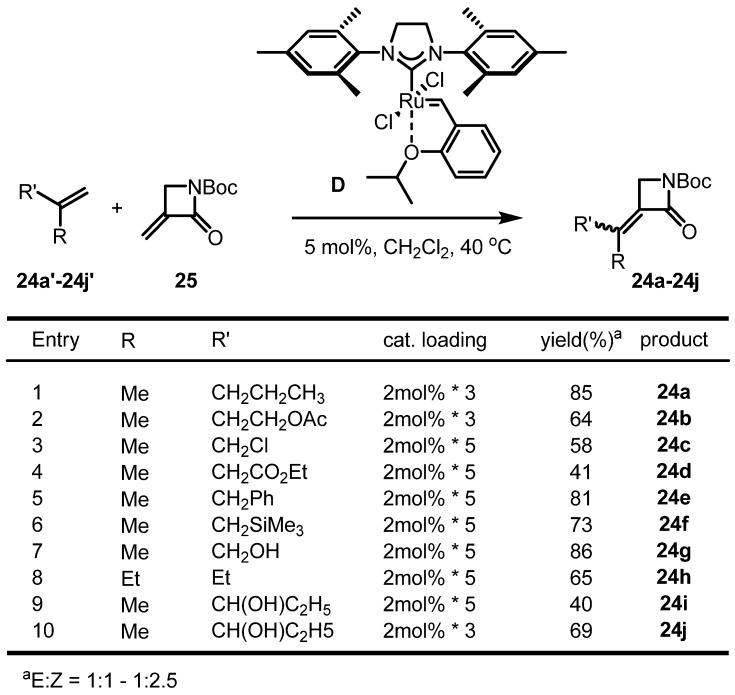
Cross metathesis in tetrasubstituted alkenes **24**.

As research on RCM in tetrasubstituted alkenes proceeds, experiments on utilizing this reaction for cross metathesis were also attempted. Howell *et al.* reported cross metathesis of the terminal alkene **24'** with β-lactam **25** to obtain the tetrasubstituted unsaturated β-lactam **24** in high yield using the Grubbs-Hoveyda catalyst **D** (Scheme 8). The reaction was quite rapid when utilized for the generation of biologically important structures with various substituents, hydroxyl, aliphatic, silyl and aromatic groups, although the olefin geometry of the product was unselective. Further results showed that the electron-rich β-lactam didn’t produce the same conversion under the same conditions, indicating that the electronic factors are important for this conversion [32].

### 2.8. Application of Metathesis in Tetrasubstituted Alkenes

**Scheme 9:**
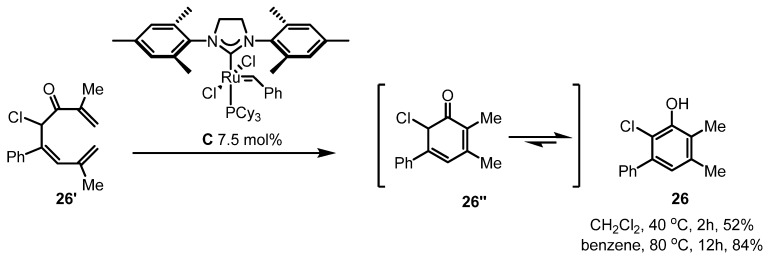
RCM in tetrasubstituted alkenes and its aromatization.

Due to little success of metathesis in tetrasubstituted alkenes, its application has been also limited. However, some results show that this conversion can be applied to the synthesis of interesting molecules when it is utilized appropriately. Actually, Yoshida *et al.* showed an example. A dimethylene compound **26'** could be converted into **26''** via the alkene metathesis reaction under the catalysis of Grubbs 2nd catalyst. Once **26''** formed, it was aromatized into the phenol **26** by spontaneous tautomerization *in situ* (Scheme 9) [33]. This reaction could be carried out in both CH_2_Cl_2_ and benzene, although the later showed better yield despite the slow reaction rate.

**Scheme 10:**
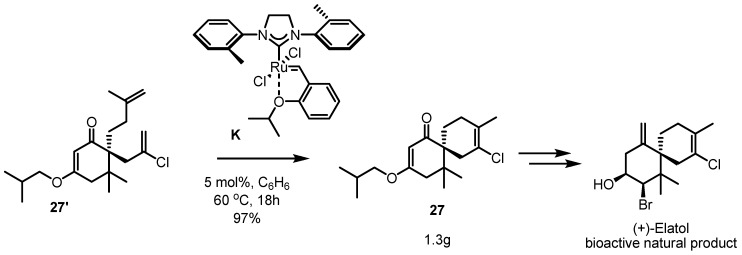
RCM in tetrasubstituted alkenes and its application to natural product synthesis.

Another application of this RCM reaction in the total synthesis of elatol, a bioactive natural product, was achieved by Stoltz *et al.* [28]. A fused bicycle **27** was produced from bis-terminal methylene **27'** through catalysis of the less sterically demanding ruthenium carbenoid **K**, described above (Scheme 10). The chloroalkene **27** could be obtained at a 97% yield and 1.3 g scale, because this procedure was so practical. It is interesting that **27** could be produced using the 2nd generation Grubbs catalyst **B** at 85% after 24 h. This intermediate **27** could be transformed into (+)-elatol via simple functional group interconversion.

These two examples show that RCM in tetrasubstituent alkenes is a highly efficient technology for the synthesis of complex molecules. More research to expand its generality is still ongoing.

## 3. Conclusions

Since the development of molybdenum and ruthenium catalysts, the metathesis reaction has served as a key carbon-carbon bond transformation. It has also allowed us to obtain otherwise unavailable tetrasubstituted alkene skeletons in an effective manner, although its substrate scope and low chemical yield hampers its wide utilization. Nowadays, however, challenges to overcome these limitations and expand its efficiency into the enantioselective reaction and the synthesis of natural products are actively ongoing. It is envisioned that this effort will result in outstanding advances in the synthesis of tetrasubstituted alkenes.
